# Comparing effects of demographic variables on current and novel neuropsychological measures in older adults with and without Alzheimer’s disease

**DOI:** 10.1002/alz.092509

**Published:** 2025-01-03

**Authors:** Alexandra M. Reed, Kevin Duff, Sydney Y Schaefer

**Affiliations:** ^1^ Arizona State University, Tempe, AZ USA; ^2^ Oregon Health & Science University, Portland, OR USA; ^3^ NIA‐Layton Aging & Alzheimer’s Disease Research Center, Portland, OR USA; ^4^ Neurosessments LLC, Tempe, AZ USA

## Abstract

**Background:**

Neuropsychological assessments are often affected by demographic factors, such as age, education, and sex. These demographic variables frequently lead to differences in test scores between minorities and non‐minorities, lowering diagnostic accuracy of Alzheimer’s disease (AD) among minoritized groups. A new test (quick Behavioral Exam to Advance Neuropsychological Screening, or qBEANS) has been developed to assess early signs of AD. The current study compared the influence of demographic variables on qBEANS and other neuropsychological tests that are commonly used in the assessment of AD.

**Method:**

In this study, 165 participants (68 cognitively unimpaired; 52 amnestic Mild Cognitive Impairment; 45 mild AD) completed the Symbol Digit Modalities Test (SDMT), Hopkins Verbal Learning Test‐Revised (HVLTR); Brief Visuospatial Memory Test‐Revised (BVMTR); and Trails Making Test (TMT) A and B. Participants also completed the qBEANS, which involves spooning two raw kidney beans at once with the nondominant hand to one of three target cups in a simple sequence, with higher scores being worse performance. Effects of age, education, and sex on raw test scores were analyzed using multivariable linear regression, with p‐values adjusted for multiple comparisons. Fisher transformations were used to examine if current tests were more affected by demographic variables than qBEANS.

**Result:**

Participants' are was 65‐91 years (mean = 74.1) and education was 12‐20 years (mean = 16), with 56.3% female. Significant age effects were observed for all tests except qBEANS (p = .10) and TMT‐A (p = .73), with older ages being associated with worse scores. Similarly, there were significant education effects for all tests except qBEANS (p = .41), TMT‐A (p = .73), and TMT‐B (p = .31), with fewer years of education being associated with worse scores. Only the HVLTR (total and delayed recall) showed a significant sex effect, with females performing better than males. The overall influence of age, education, and sex on qBEANS scores was significantly smaller than the SDMT, HVLTR, and BVMTR (no different with TMT‐A or TMT‐B).

**Conclusion:**

Unlike most neuropsychological tests, qBEANS may be appropriate for screening diverse populations without controlling for demographic variables. Future research should consider populations with lower educational attainment, as well as race, ethnicity, and native language on qBEANS relative to other neuropsychological measures.

